# Green and sustainable catalyst-free synthesis of 2-benzylidene-indan-1,3-dione derivatives using concentrated solar radiation in polyethylene glycol

**DOI:** 10.1039/d5ra08259e

**Published:** 2026-02-05

**Authors:** Milad Ahmadi, Ali Reza Kiasat, Mohammad Sabaeian, Mohammad Reza Dayer

**Affiliations:** a Chemistry Department, College of Science, Shahid Chamran University of Ahvaz Ahvaz Iran milad137651821@gmail.com +98 937 536 5401; b Department of Physics, Faculty of Science, Shahid Chamran University of Ahvaz Ahvaz Iran; c Center for Research on Laser and Plasma, Shahid Chamran University of Ahvaz Ahvaz Iran; d Department of Biology, Faculty of Science, Shahid Chamran University of Ahvaz Ahvaz Iran

## Abstract

The increasing demand for sustainable and environmentally friendly synthetic methodologies has driven the development of catalyst-free and energy-efficient organic transformations. In this study, we report a green and practical protocol for the synthesis of 2-benzylidene-indan-1,3-dione (BZI) derivatives *via* Knoevenagel condensation of aromatic aldehydes and 1*H*-indene-1,3(2H)-dione under concentrated solar radiation (CSR), a clean, renewable, and abundant energy source. The reactions were carried out in polyethylene glycol-400 (PEG-400), a biodegradable, non-volatile, and recyclable solvent, under mild, catalyst- and additive-free conditions. The method affords good to excellent isolated yields (74–98%) in significantly reduced reaction times, eliminating the use of hazardous reagents and minimizing waste generation. Mechanistic investigations using 2,2-diphenyl-1-picrylhydrazyl (DPPH) and 2,2,6,6-tetramethylpiperidin-1-yl)oxyl (TEMPO), two well-known radical scavengers, confirmed a non-radical, photo-thermal activation pathway driven by synergistic effects of the UV and IR components of solar radiation and the solvation properties of PEG-400. Green chemistry metrics were calculated for the model reaction, revealing an Atom Economy of 85.4%, Carbon Efficiency of 100%, low E-factor (0.55), and Reaction Mass Efficiency (RME) of 70.3%. This simple, cost-effective, and eco-friendly protocol aligns well with the principles of green chemistry, presenting a scalable alternative for the sustainable synthesis of BZI derivatives under environmentally benign conditions.

## Abstract 2

The increasing demand for sustainable and environmentally friendly synthetic methodologies has driven the development of catalyst-free and energy-efficient organic transformations.

## Graphical abstract

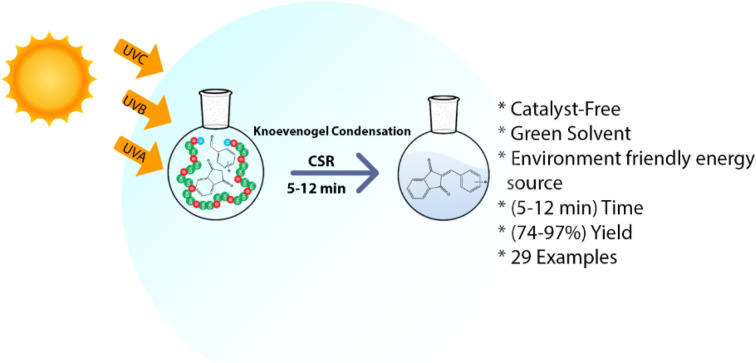

## 1. Introduction

In recent years, green chemistry has evolved into a foundational framework in contemporary synthetic chemistry, advocating the development of environmentally benign, energy-efficient, and atom-economical processes. Central to this discipline is the design of chemical reactions that avoid the use and generation of toxic substances—both in reactants and solvents—while promoting safer alternatives. Key principles include minimizing waste, improving reaction selectivity and efficiency, lowering energy requirements, and utilizing renewable feedstocks or benign reaction media. These priorities have become particularly significant in response to the growing demand for sustainable technologies in both academic research and industrial applications.^1,2^ The well-recognized environmental and health risks associated with conventional organic solvents, along with difficulties in catalyst separation and recyclability, have spurred the development of greener, scalable, and more sustainable synthetic protocols. These methodologies are in accordance with the fundamental principles of green chemistry, emphasizing waste minimization, the avoidance of toxic reagents, and the achievement of high product yields under milder conditions and shorter reaction times.^3,4^ The accelerating global population growth and industrial expansion have led to a marked increase in the consumption of non-renewable resources, especially fossil fuels, resulting in elevated emissions of hazardous pollutants, including greenhouse gases. These emissions pose serious risks to ecosystems and public health. Confronting this challenge requires a paradigm shift toward the development and implementation of renewable, low-impact alternatives in chemical synthesis and industrial processes to reduce environmental burdens and support long-term ecological sustainability.^5,6^ The use of renewable energy sources that are both environmentally benign and economically viable is crucial for the advancement of sustainable chemical processes.^7^ To achieve this goal, various energy-efficient techniques such as microwave irradiation (MW), ultrasonication, mechanochemistry, and ultraviolet (UV) irradiation have been extensively investigated and applied.^8,9^ Nevertheless, the dependence on costly specialized equipment and skilled personnel poses significant challenges to the scalability and sustainable implementation of these techniques. Therefore, there is an urgent need to develop practical, environmentally benign, and economically feasible alternatives.

Recent studies in sustainable organic synthesis have emphasized the use of clean energy, such as visible light and electricity, to drive chemical transformations under mild and eco-friendly conditions. Approaches including electrified high-temperature methods, photoelectrocatalysis, and visible-light photoredox catalysis enable efficient, low-carbon, and selective functionalizations.^10–12^ These advances highlight the value of integrating renewable energy into synthesis, providing context for the solar-driven transformations explored in this study.

2-Benzylidene-indan-1,3-dione (BZI) derivatives have attracted significant interest due to their diverse biological activities and potential applications in the synthesis of pharmacologically relevant compounds.^13^ These compounds demonstrate notable antioxidant, anticancer, antibacterial, antiviral, antifungal, and enzyme inhibitory properties, particularly against lipoxygenase, tyrosinase, and urease enzymes.^14–20^ Considering their promising therapeutic profiles, the development of straightforward, cost-effective, and environmentally benign synthetic methods for BZI derivatives remains a compelling objective.

Solar energy is widely acknowledged as one of the most sustainable, abundant, and readily available energy sources, offering a clean, cost-effective, and environmentally benign alternative to conventional fossil fuels.^21^ As the Earth's primary and inexhaustible energy input, solar radiation delivers an estimated intensity of approximately 120 petawatts at the surface, sufficient to meet global energy demands for over two decades.^22,23^ Solar radiation encompasses UV, visible, and infrared (IR) photons, covering wavelengths from about 100 to 1 000 000 nm. While IR radiation mainly contributes to thermal energy, UV and visible photons are integral to photoexcitation processes driving diverse chemical transformations. Despite its immense potential, nearly 1.3 billion people—around 13% of the world's population—lack access to electricity, restricting their ability to fulfill basic needs. In response, solar-driven chemical synthesis methods have been developed as sustainable alternatives to conventional electricity-dependent processes.^24,25^ Sunlight concentrators, such as solar reflectors, have recently been developed to enable high-temperature chemical transformations driven exclusively by focused solar irradiation.^26^ A variety of organic reactions have been successfully conducted under concentrated solar radiation (CSR), including the synthesis of *N*-phenylphthalimide,^24^ 3-alkylated indole derivatives,^25^ dihydropyridines,^27^ Heck coupling reactions,^28^ alcohol oxidations,^29^ and benzylic brominations.^30^

Various synthetic methods have been reported for the preparation of BZI derivatives, employing catalysts such as L-proline,^31^ piperidine,^15^ and zirconyl chloride octahydrate,^17^ in both polar and non-polar solvents including methanol,^31^ dichloromethane,^32^ and toluene.^33^ However, many of these protocols suffer from drawbacks including the use of expensive or toxic catalysts, volatile and hazardous solvents, and harsh reaction conditions. Accordingly, the development of novel catalyst-free synthetic approaches utilizing green solvents has garnered significant attention.^34–36^

Polyethylene glycol (PEG) has emerged as an attractive, environmentally benign, and cost-effective solvent in organic synthesis. Its favorable properties—including biocompatibility, low toxicity, affordability, recyclability, biodegradability, and excellent thermal stability—have established PEG as a preferred reaction medium for various green transformations. Notably, numerous carbon–carbon bond-forming reactions, such as Heck, Stille, Suzuki, Sonogashira, and Hiyama couplings, have been successfully conducted in PEG-based systems.^37–45^

**Fig. 1:**
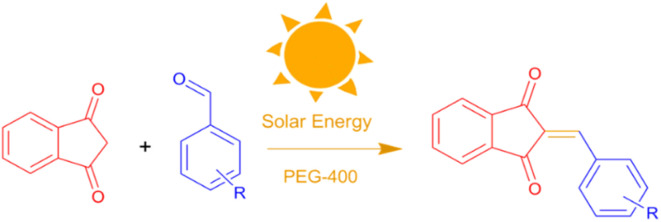
Catalyst-free synthesis of BZI derivatives in PEG-400 under CSR.

In this context, the present study introduces a novel, sustainable, and catalyst-free protocol for the synthesis of BZI derivatives *via* a Knoevenagel condensation reaction driven by CSR. The reaction is carried out in polyethylene glycol 400 (PEG-400), a biodegradable, recyclable, and non-toxic solvent, under green and additive-free conditions. This environmentally benign method avoids the use of hazardous reagents and expensive catalysts, providing a simple, low-cost, and efficient strategy for C=C bond formation. As illustrated in Fig. 1, the proposed approach is in strong alignment with the key principles of green chemistry, offering high atom economy, operational simplicity, and promising scalability for sustainable organic synthesis.

## 2. Material and methods

### 2.1 General

All chemicals and solvents were purchased from Merck (Germany) and used without further purification. Reaction progress was monitored by thin-layer chromatography (TLC) on aluminum-backed silica gel 60 F_254_ plates under UV light. Melting points were determined using the capillary method on an Electrothermal 9200 apparatus and are reported uncorrected. Infrared spectra (FT-IR) were recorded on a Thermo Scientific Nicolet iS10 spectrometer using KBr pellets, and absorption bands are expressed in cm^−1^. ^1^H NMR spectra were acquired on a Bruker Avance DPX 250 MHz spectrometer in DMSO-d_6_ with tetramethylsilane (TMS) as the internal reference. Solar irradiance (W m^−2^) was measured using a calibrated TES Solar Power Meter (Model 1333, Taiwan), and the reaction temperatures were continuously monitored with a digital infrared thermometer (Non Per Umani; −50 to 550 °C). All solar-assisted reactions were conducted under direct sunlight between 10:00 AM and 12:30 PM in Ahvaz, Iran, corresponding to the period of maximum solar intensity. Experiments were carried out outdoors at the Faculty of Science, Shahid Chamran University of Ahvaz, Khuzestan, Iran (31°00′30″ N, 48°39′57″ E; 20 m above sea level).

### 2.2 CSR assembly

**Fig. 2:**
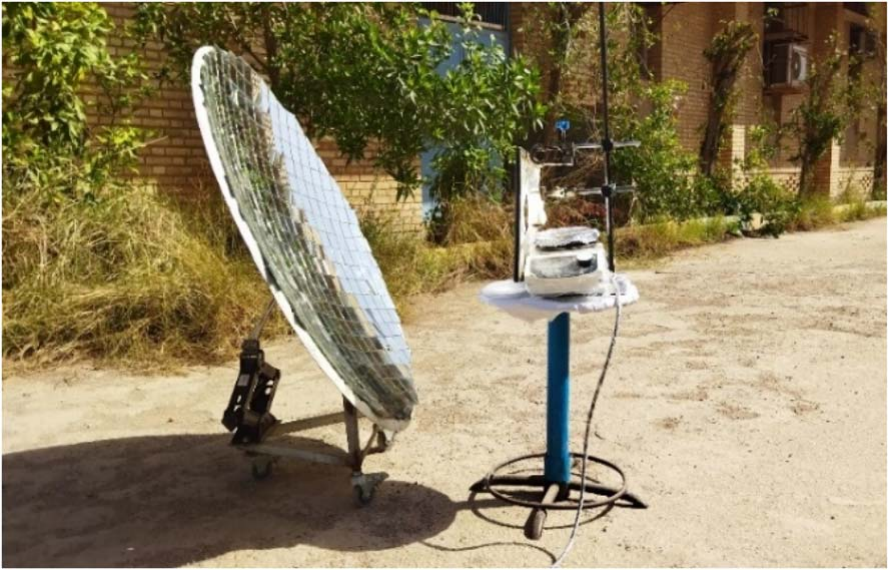
CSR assembly used for the synthesis of BZI derivatives.

**Fig. 3:**
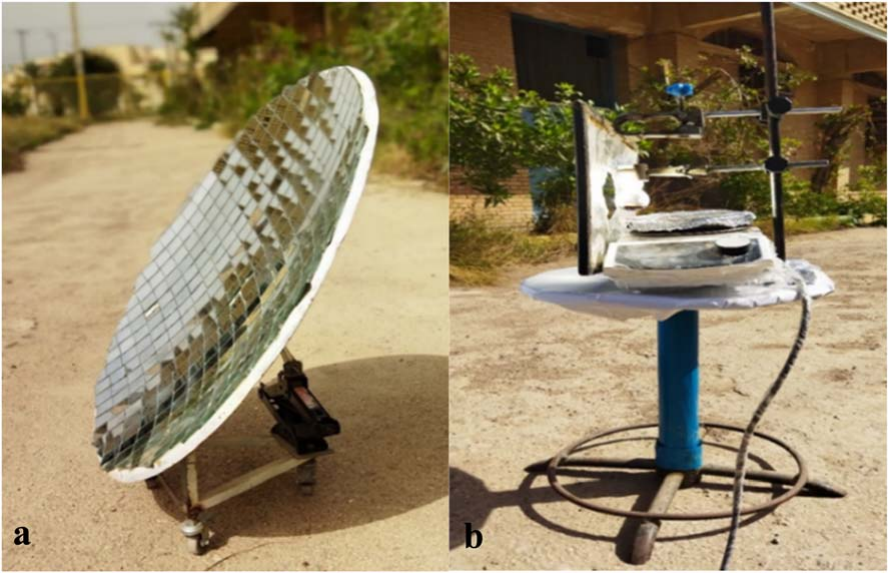
Components of the CSR system: (a) reflector and (b) turret used for synthesizing BZI derivatives.

The synthesis of BZI derivatives was carried out using a custom-designed CSR system, as shown in Fig. 2. The setup featured an 80 cm parabolic dish reflector (PDR) covered with 4 × 4 cm square mirrors coated with mercury amalgam to enhance reflectivity. Mounted on a rhombus jack with caster wheels, the system allowed dual-axis solar tracking and precise focusing. The turret assembly (Fig. 3) included a magnetic stirrer, aluminum platform, and adjustable clamps for stable positioning of the reaction vessel. The apparatus could rotate 180° to maintain continuous solar alignment and maximize energy capture during the reaction.

### 2.3 CSR-promoted synthesis of BZI derivatives

In a typical procedure, a round-bottom flask containing 1.0 mmol of 1*H*-Indene-1,3(2H)-dione, 1.2 mmol of the respective aromatic aldehyde, and 2 mL of PEG-400 was placed on a magnetic stirrer at the focal point of the parabolic dish reflector. The reaction mixture was stirred under concentrated solar radiation until completion, as monitored by thin-layer chromatography (TLC). Upon completion, the mixture was allowed to cool, and 10 mL of distilled water was added to precipitate the product. The solid was collected by filtration and purified by recrystallization, affording the desired 2-benzylidene-indan-1,3-dione derivatives in isolated yields ranging from 74% to 98% (Table 4). Reaction temperatures, monitored by a digital infrared thermometer, ranged from 95 to 160 °C, while ambient temperatures varied between 33 and 42 °C. Peak solar irradiation intensity, measured using a calibrated solar power meter, was recorded between 850 and 1000 W m^−2^.

### 2.4 Green chemistry metrics

To evaluate the environmental efficiency of the CSR-assisted synthesis of 2-benzylidene-indan-1,3-dione derivatives, several green chemistry metrics were calculated, including Atom Economy (AE), E-factor, Carbon Efficiency (CE) and Reaction Mass Efficiency (RME).^43^

Atom Economy (AE) was calculated using the formula:
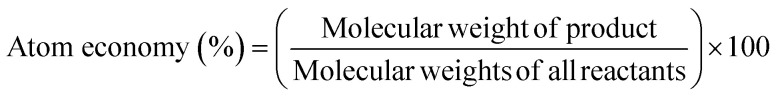


E-factor, which quantifies the amount of waste generated per unit mass of product, was calculated as:
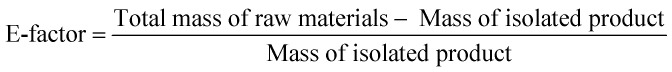


Reaction Mass Efficiency (RME), reflecting the proportion of reactant mass converted into the desired product, was calculated by:
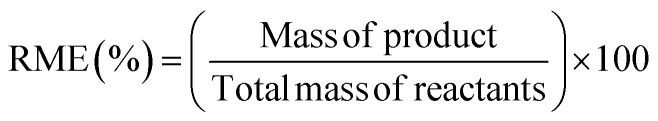


Carbon Efficiency (CE%) was also calculated to assess the retention of carbon atoms from reactants in the final product. The CE was determined using the formula:
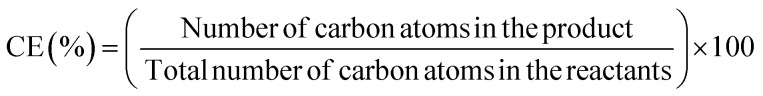


The isolated product mass was recorded as 221.252 mg.

### 2.5 Selected spectra data

#### 2.5.1 2-(2-hydroxy- 4-methoxybenzylidene)-2*H*-indene-1, 3-dione (4m)

Green solid; mp: 184–185 °C: IR (*ν*_max/cm^−1^, KBr): 3331, 3099, 2932, 2833, 1719, 1678, 1603, 1578, 1481, 1388, 1246, 1222, 1148, 1099, 1024, 777, 750, 737, 626, 545, 529, 421; ^1^H NMR (250 MHz, DMSO–d_6_): *δ* = 10.58 (s, 1H), 8.67 (s, 1H), 7.89 (s, 5H), 7.73 (s, 1H), 6.94–6.90 (d, 1H), 3.90 (s, 3H).

#### 2.5.2 2-(4-chlorobenzylidene)-2*H*-indene-1, 3-dione (4 t)

Yellow solid; mp: 173–175 °C: IR (*ν*_max/cm^−1^, KBr): 3092, 3060, 1775, 1726, 1691, 1611, 1589, 1557, 1431, 1381, 1354, 1246, 1203, 1092, 991, 831, 780, 735, 531, 509, 446; ^1^H NMR (250 MHz, DMSO–d6): *δ* = 8.43–8.40 (d, 2H), 7.89 (s, 4H), 7.72 (s, 1H), 7.55–7.51 (d, 2H).

#### 2.5.3 2-(4-aminobenzylidene)-2*H*-indene-1,3-dione (4x)

Red solid; mp: 262–263 °C: IR (*ν*_max, KBr): 3331, 3099, 2932, 2833, 1719, 1678, 1603, 1578, 1481, 1388, 1246, 1222, 1148, 1099, 1024, 777, 750, 737, 626, 545, 529, 421; ^1^H NMR (250 MHz, DMSO–d_6_): *δ* = 8.43–8.40 (d, 1H), 7.81 (s, 2H), 7.58 (s, 5H), 6.96 (s, 1H), 6.68–6.64 (d, 2H).

## 3. Results and discussion

### 3.1 Reaction optimization and solvent effect

In response to the increasing demand for sustainable and environmentally benign synthetic methodologies, we investigated the use of CSR in combination with PEG-400 as a green, biodegradable, and catalyst-free reaction medium for the synthesis of BZI derivatives. CSR provides a dual energy input, delivering both thermal energy and UV photons, which together enable concurrent thermal and photochemical activation of the reaction.^46^ Additionally, the IR component of solar radiation enhances molecular vibrational energy, thereby increasing the frequency and efficacy of molecular collisions. PEG-400, characterized by its high thermal stability, low toxicity, and excellent solubilizing properties, serves not only as a benign solvent but also promotes substrate proximity through its flexible polyether chains, acting as a pseudo-catalyst.^47^ The synergistic interplay between CSR and PEG-400 thus facilitates a straightforward, energy-efficient, and catalyst-free approach to synthesize BZI derivatives under environmentally friendly conditions.

To optimize the reaction parameters, the condensation of 1*H*-indene-1,3(2H)-dione and 4-chlorobenzaldehyde in PEG-400 was selected as the model system (Table 1). A systematic comparison was conducted using various energy sources—including conventional thermal heating, MW irradiation, ultrasonic activation, and CSR—under identical catalyst-free conditions. Among these, CSR significantly outperformed other energy inputs, affording the target product in a remarkably shorter time (10 min) and with a commendable yield (81%). In contrast, conventional heating required prolonged durations (up to 150 min), and MW-assisted synthesis resulted in moderate yields. Mechanical grinding, visible light, UV irradiation (256 nm), and ambient conditions all proved insufficient to drive the reaction to completion within reasonable timeframes. The superior performance of CSR is attributed to the synergistic interaction between UV and IR components of sunlight: UV photons initiate photoexcitation of reactive species, while IR radiation enhances thermal energy input, thereby increasing molecular vibration and collision frequency. This dual activation mechanism facilitates efficient C=C bond formation and underscores the potential of CSR as a clean, sustainable, and cost-effective energy source for green organic transformations.

**Table 1:** Comparison of different energy sources for the synthesis of BZI by condensation of 1*H*-indene-1,3(2H)-dione (1 mmol) and 4-chlorobenzaldehyde (1.2 mmol) in 2 mL of solvent

Entry	Energy source/method	Temperature (°C)	Time (min)	Irradiance/power	Wavelength	Yield (%)
1	CSR	136	10	888 W m^−2^	Natural sunlight	81
2	Conventional thermal heating (85 °C)	85	150	—	—	88
3	Conventional thermal heating (140 °C)	140	80	—	—	84
4	MW	100	15	1800 W	1.22 × 10^8^ nm	68
5	Ultrasonic bath	70	120	—	—	75
6	Grinding	RT	180	—	—	Incomplete reaction
7	Visible light	RT	360	32 W	400–700 nm	Incomplete reaction
8	UV (256 nm)	RT	360	32 W	256 nm	Incomplete reaction
9	Room temperature	RT	1440	—	—	Incomplete reaction

To further refine the reaction system, the influence of various solvents was examined under CSR irradiation (Table 2). Both polar protic and aprotic solvents were evaluated—including water, isobutanol, cyclohexanol, DMSO, DMF, PEG-300, PEG-400, and diethylene glycol. Among the tested solvents, PEG-400 demonstrated the best performance, achieving the highest yield in the shortest time (10 min, 81% yield). This enhanced efficiency can be attributed to PEG-400's high boiling point, thermal stability, and strong solvation ability, which collectively support effective reactant solubilization and energy transfer. Moreover, PEG-400 aligns with green chemistry principles due to its low toxicity, biodegradability, and recyclability, reinforcing its role as an environmentally favorable medium for sustainable synthesis.

**Table 2:** Effect of solvents on the catalyst-free synthesis of BZI by condensation of 1*H*-indene-1,3(2H)-dione (1 mmol) and 4-chlorobenzaldehyde (1.2 mmol) in 2 mL of solvent under CSR

Entry	Solvent	Time (min)	Yield (%)
1	Water	20	50
2	DMSO	15	66
3	DMF	15	68
4	Isobutanol	15	70
5	Cyclohexanol	20	77
6	PEG-400	10	81
7	PEG-300	15	62
8	Diethylene glycol	30	56

### 3.2 Effect of solar intensity and seasonal variations

Given the inherent reliance of CSR efficiency on solar intensity, the model Knoevenagel condensation reaction was evaluated under different seasonal conditions to assess the impact of natural solar variability on reaction performance (Table 3). The data clearly demonstrate that the synthesis proceeds efficiently during spring and summer months, when high ambient temperatures and strong solar irradiance prevail. Under these favorable conditions, the reaction was completed within 10 minutes, yielding 2-benzylidene-indan-1,3-dione with 81% isolated yield. In contrast, reduced sunlight exposure in autumn (November) extended the reaction time to 15 minutes and slightly decreased the yield to 78%. Notably, under winter conditions (December), characterized by significantly lower ambient temperatures (20 °C) and minimal solar irradiance (172 W m^−2^), the reaction did not proceed to completion. These results validate the seasonal adaptability and limitations of CSR-based synthesis, emphasizing the importance of optimizing solar collection strategies or integrating auxiliary energy inputs during low-irradiance periods for consistent year-round performance.

**Table 3:** Seasonal variations in the CSR-promoted synthesis of BZI derivatives (1*H*-indene-1,3(2H)-dione, 1 mmol; 4-chlorobenzaldehyde, 1.2 mmol; solvent, 2 mL)

	31 May	11 July	1 November	2 December
Reaction initiation time	10 min	10 min	15 min	Incomplete reaction
Average outside temperature	38 °C	41 °C	30 °C	20 °C
Outside conditions	Sunny	Sunny	Partly cloudy	Cloudy
Average solar irradiation (w m^−2^)	887	940	675	172
Isolated yield (%)	81	81	78	0

**Fig. 4:**
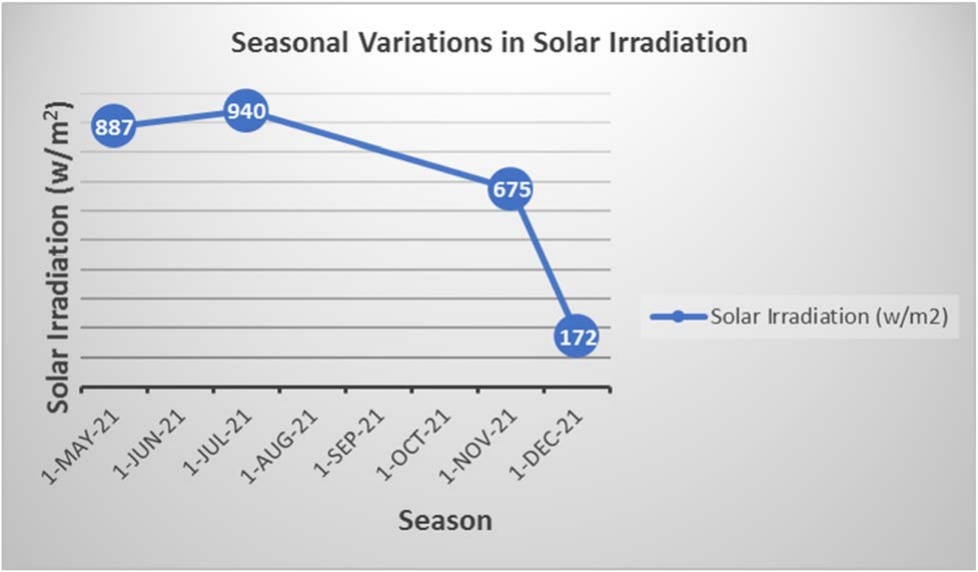
Seasonal variations in solar radiation intensity.

Analysis of solar irradiance data (Fig. 4) reveals that a minimum solar intensity threshold of approximately 600 W m^−2^ is generally required to supply adequate thermal energy for efficient progression of the reaction. Solar intensities above this threshold correlate with rapid reaction initiation and high product yields under CSR conditions. In contrast, reduced irradiance—particularly under overcast skies and lower ambient temperatures typical of autumn and winter—significantly diminishes the reaction rate and overall efficiency. These findings underscore the critical influence of both solar intensity and environmental conditions on the viability of CSR-driven organic transformations, highlighting the need for adaptive operational strategies in less favorable climates.

### 3.3 Substrate scope and yields

Encouraged by these promising results, we explored the generality of the CSR-assisted protocol by extending the substrate scope to a series of aromatic aldehydes bearing diverse functional groups (Fig. 1 and Table 4). The results demonstrated that the Knoevenagel condensation proceeded smoothly under catalyst-free conditions, with both electron-donating and electron-withdrawing substituents being well tolerated. This broad functional group compatibility underscores the robustness of the method. Yields of the desired BZI derivatives ranged from moderate to excellent, and all products were fully characterized by comparison of their melting points and spectroscopic data (IR and ^1^H NMR) with previously reported literature values, confirming the structures unambiguously.

**Table 4:** CSR-assisted synthesis of 2-benzylidene-1*H*-indene-1,3(2H)-dione derivatives in PEG-400

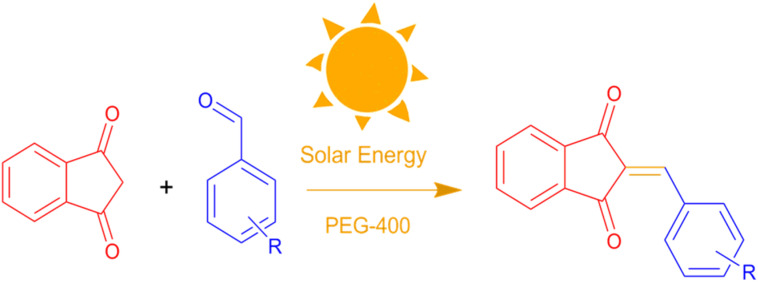					
Entry	R	Product	Time (min)	Yield (%)	MP (°C) found (reported)
1	H	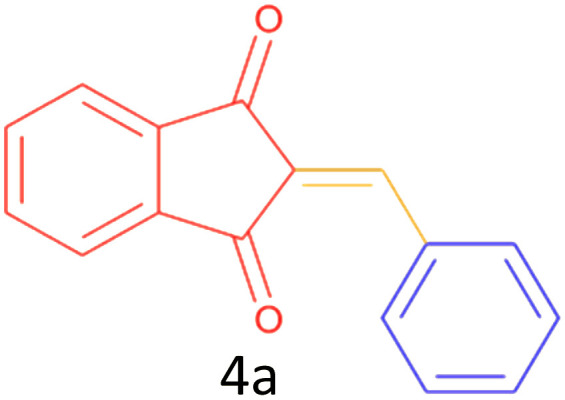	10	77	150–152(149–150)^48^
2	2-NO_2_	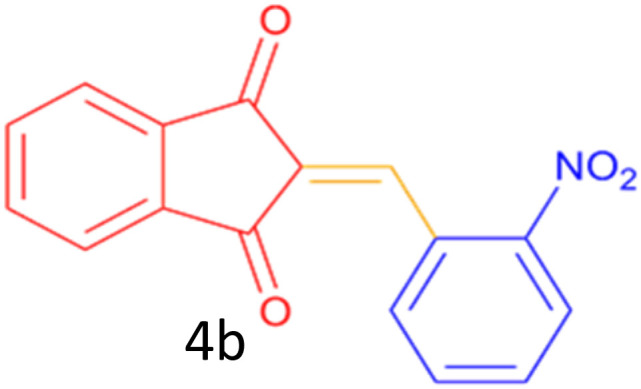	5	91	189–190(190)^49^
3	3-NO_2_	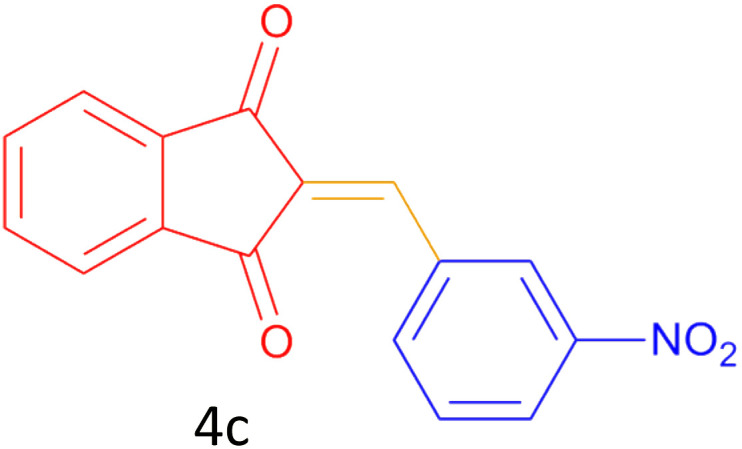	5	93	246–247(243–246)^50^
4	4-NO_2_	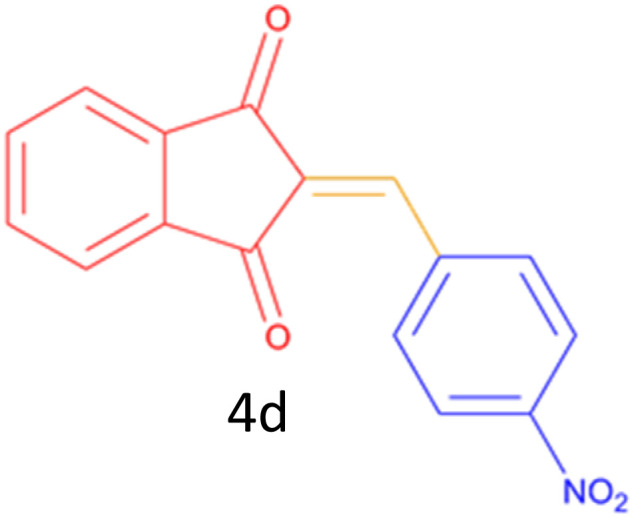	5	94	235–237(234–236)^13^
5	4-Cl,3-NO_2_	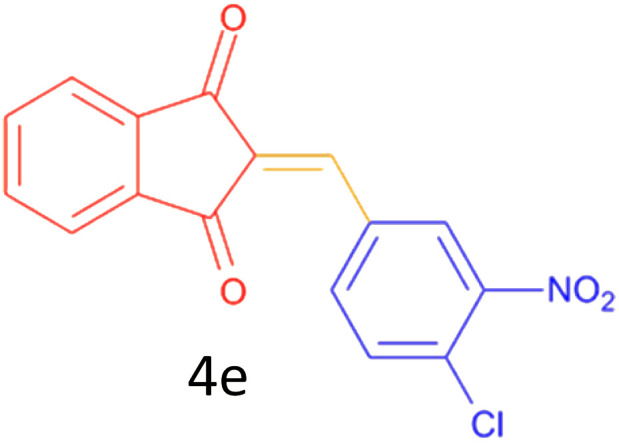	5	97	227–229(222–224)^51^
6	4-CN	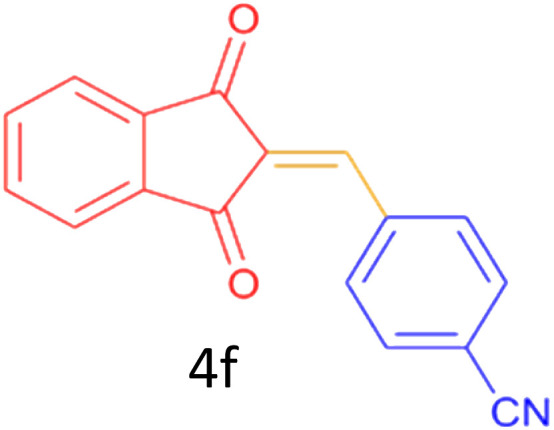	5	92	239–240(238–240)^49^
7	2-OH	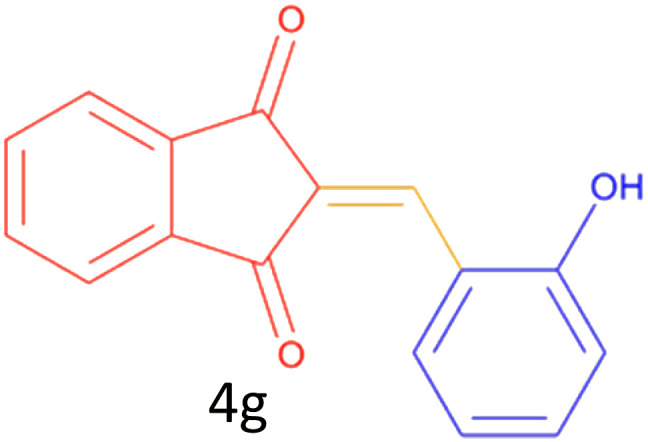	10	92	202–203(198.6–199.3)^52^
8	3-OH	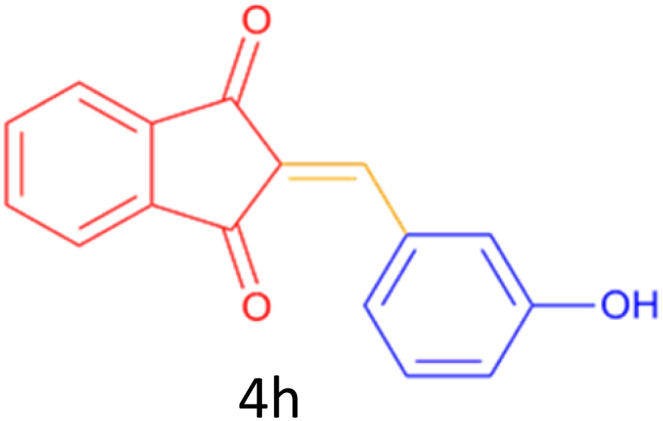	10	80	217–219(219–222)^50^
9	4-OH	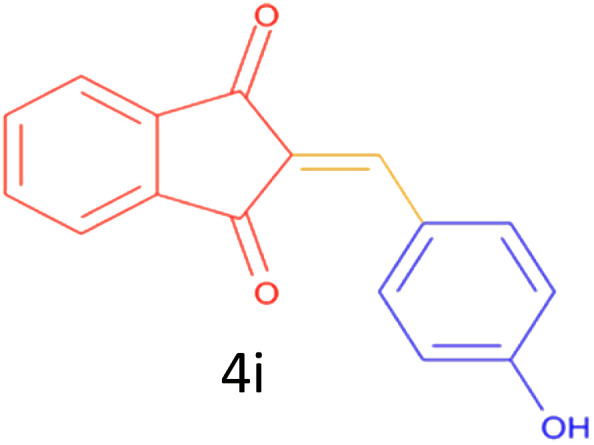	10	82	239–240(241–243)^13^
10	2,4-diOH	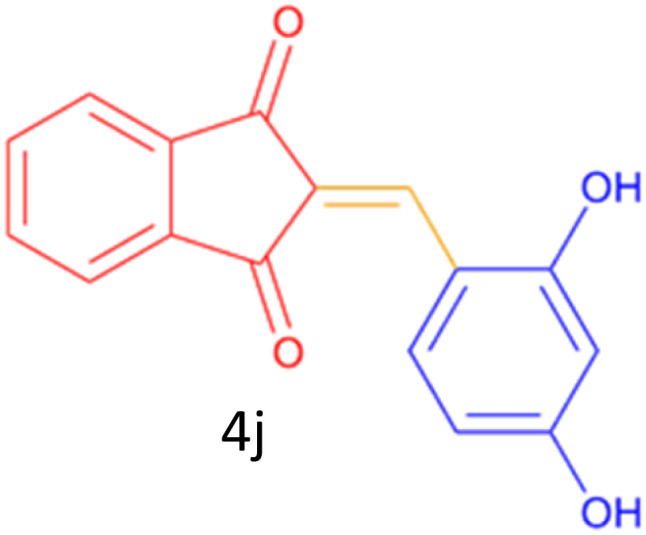	10	88	233–234(232.4)^53^
11	3,4-diOH	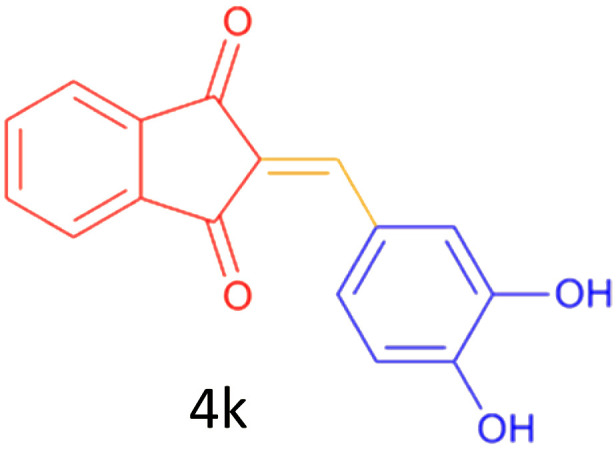	10	86	245–247(250)^53^
12	2-OH,3-OMe	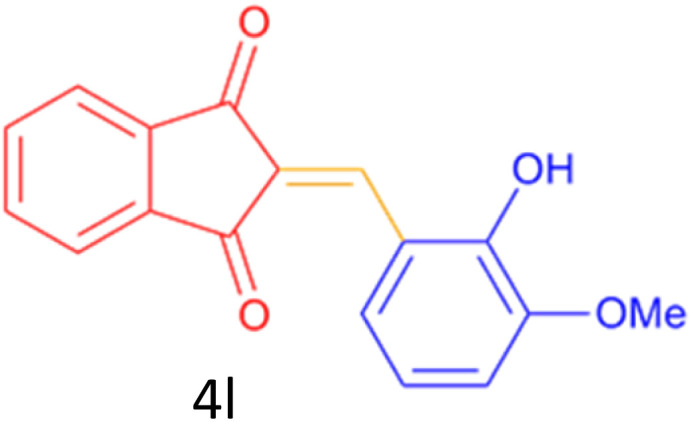	10	88	228–229(225)^53^
13	2-OH,4-OMe	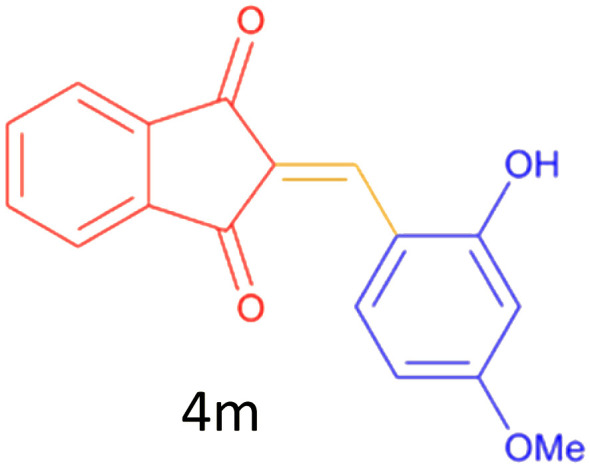	10	85	184–185(176–180)^20^
14	2-OH,5-Br	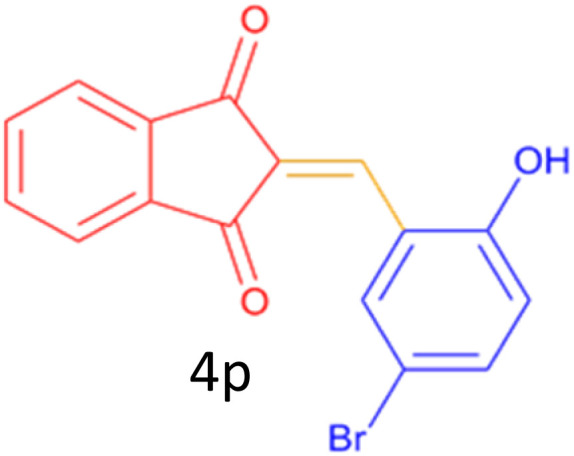	5	98	222–224(220.5)^54^
15	2-Cl	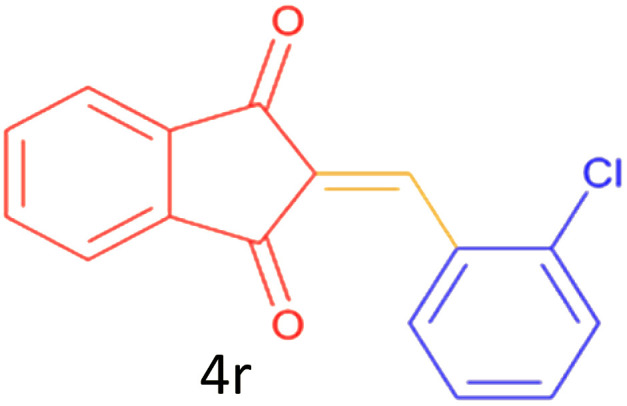	10	79	132–133(132–133)^48^
16	3-Cl	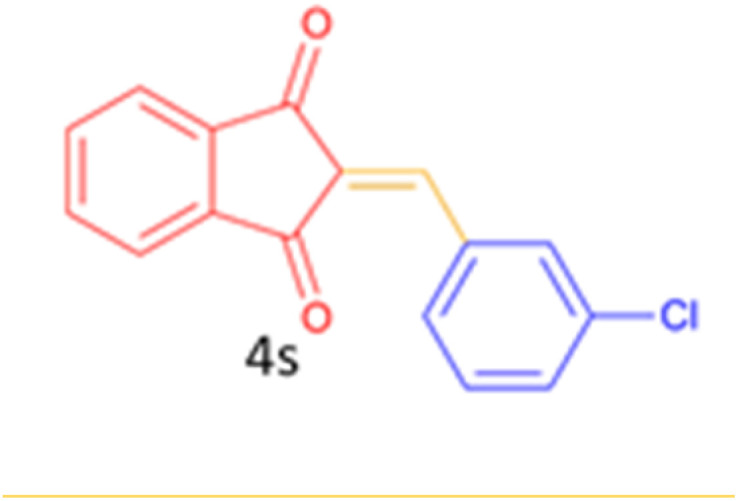	10	81	165–167(167–168)^48^
17	4-Cl	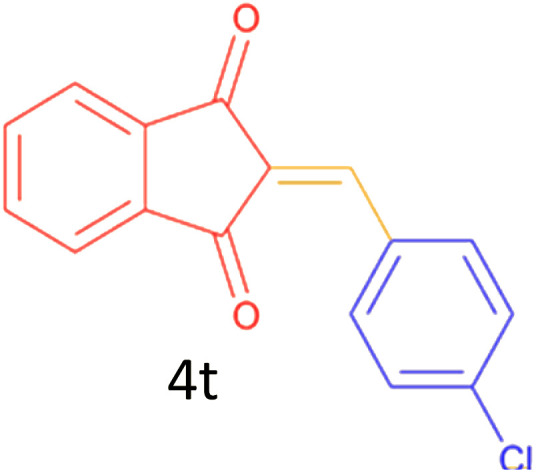	10	81	173–175(172–174)^48^
18	3,4-diCl	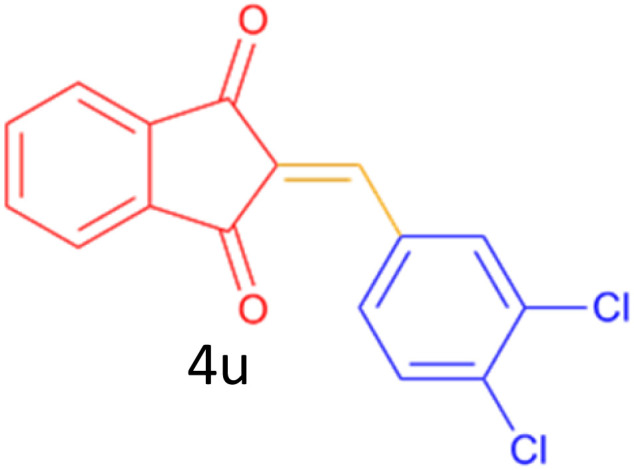	12	77	238–239(230–231)^55^
19	4-Br	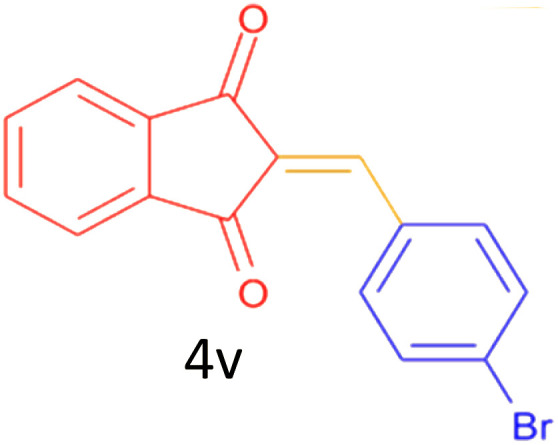	10	80	170–172(173–175)^49^
20	3-F	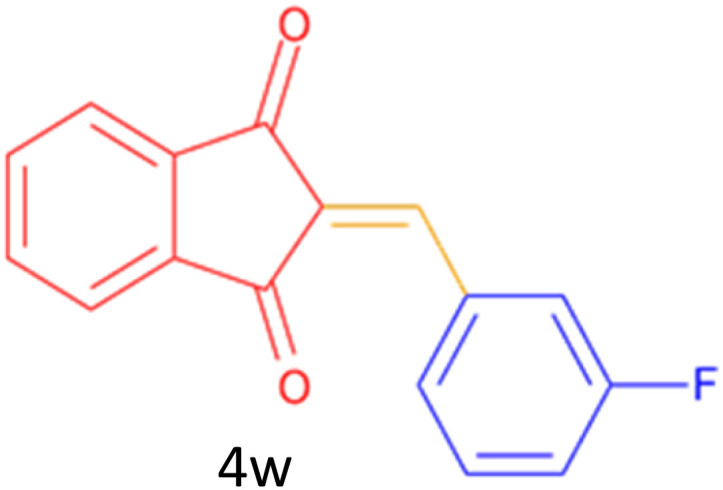	10	77	169–171(175)^56^
21	4-NH_2_	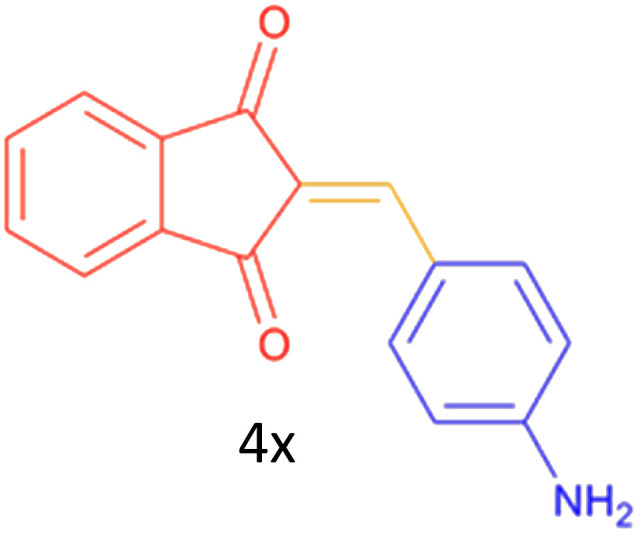	10	97	262–263(261–262)^57^
22	4-N(Me)2	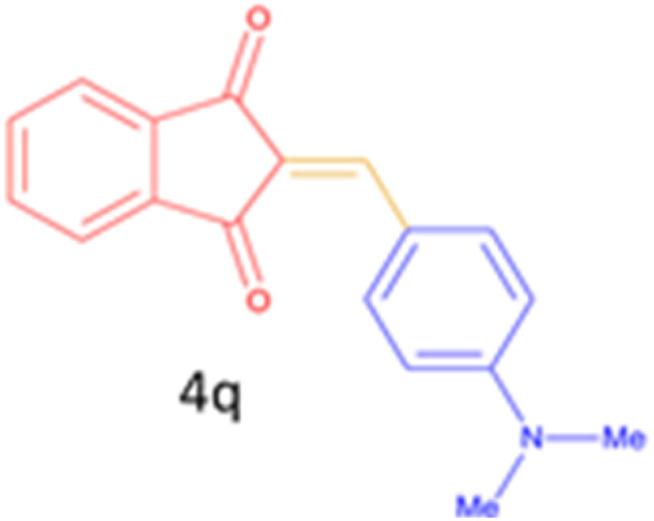	15	80	180–181(180.1–181.5)^17^
23	3-OMe	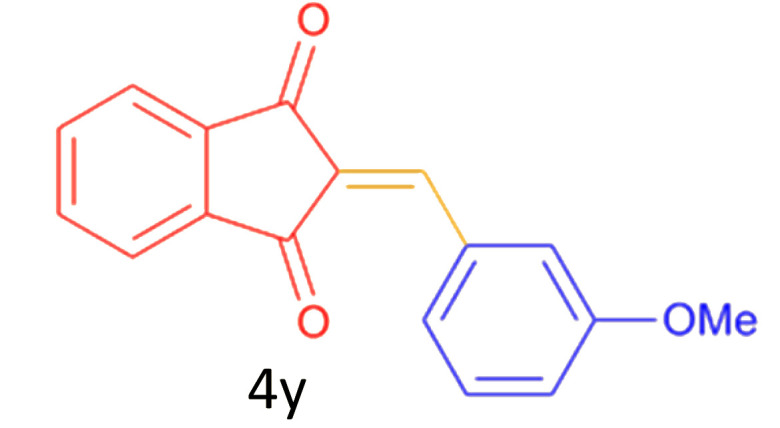	12	78	140–142(139–141)^50^
24	4-OMe	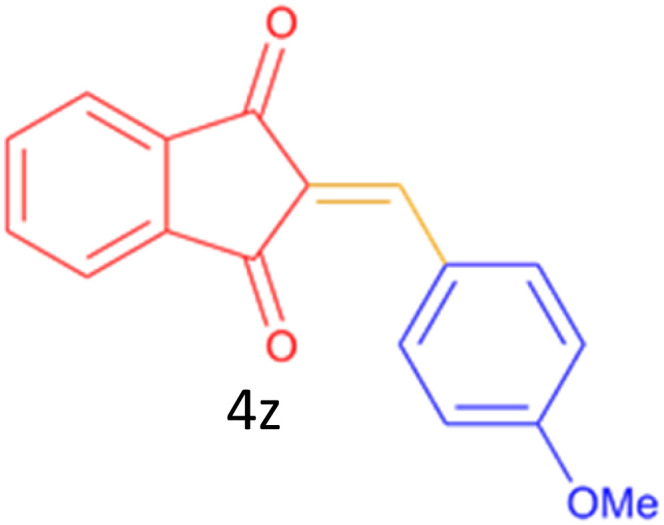	12	80	152–154(152–155)^50^
25	4-Me	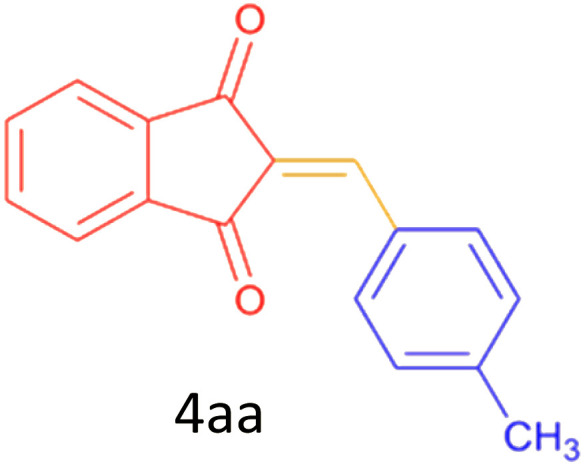	12	77	148–150(150–151)^48^
26	2-Thiophene carboxaldehyde	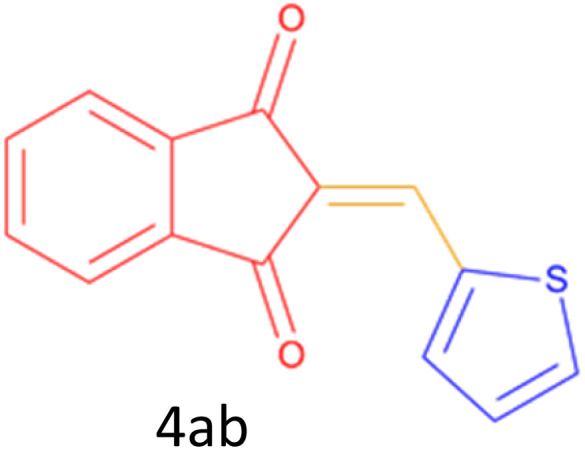	5	76	162–164(165–166)^58^
27	4-Pyridine carboxaldehyde	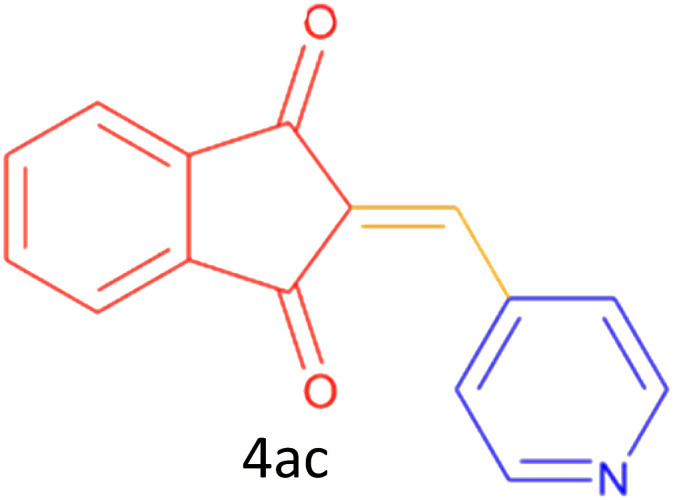	5	74	164–165(167)^59^
28	4-CHO (1 mmol indandion)	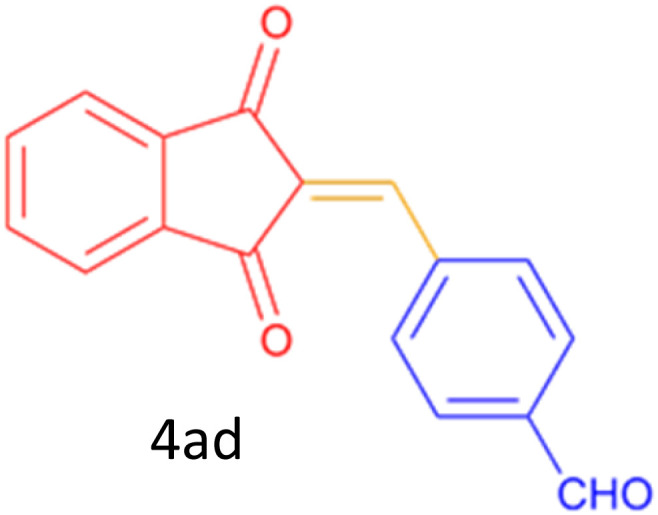	5	93	171–172(173)^60^
29	4-CHO (2 mmol indandion)	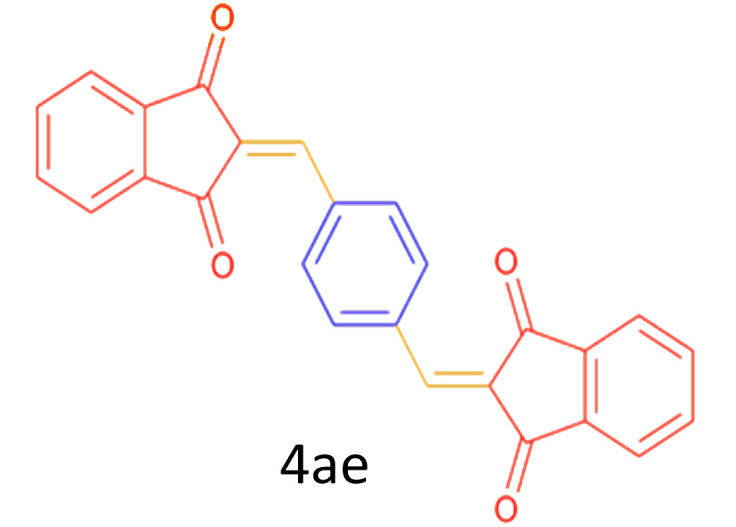	5	91	360–362(360–362)^61^

### 3.4 Mechanistic studies

**Fig. 5:**
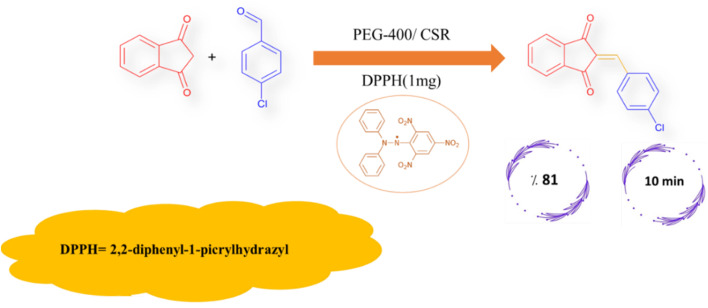
Evaluation of the reaction pathway under CSR conditions in PEG-400 using DPPH as a radical scavenger, indicating a photo-thermal rather than photo-radical mechanism.

To elucidate whether the CSR-assisted Knoevenagel condensation proceeds *via* a photo-thermal or photo-radical mechanism, the model reaction between 1*H*-indene-1,3(2H)-dione and 4-chlorobenzaldehyde was carried out in PEG-400 under CSR in the presence of 2,2-diphenyl-1-picrylhydrazyl (DPPH), a well-known radical scavenger (Fig. 5). The reaction was also performed in the presence of another radical scavenger, 2,2,6,6-tetramethylpiperidin-1-yl)oxyl (TEMPO), at varying amounts (1, 5, 10, and 20 mg, Table 5). As illustrated in Fig. 5, the reaction proceeded smoothly, affording 2-(4-chlorobenzylidene)-1*H*-indene-1,3(2H)-dione in 81% yield within 10 min, consistent with the reaction performed in the absence of radical scavengers. As summarized in Table 5, neither the reaction time nor the product yield was affected by the presence of these scavengers. These results clearly indicate that radical intermediates are not involved in this transformation, confirming that the reaction proceeds predominantly *via* a photo-thermal activation pathway under CSR conditions.

**Table 5:** Effect of radical scavengers on CSR-assisted Knoevenagel condensation

Entry	Scavenger	Amount (mg)	Time (min)	Yield (%)	Observation
1	None	0	10	81	Standard reaction, no scavenger
2	DPPH	1	10	81	No change
3	DPPH	5	10	81	No change
4	DPPH	10	10	81	No change
5	DPPH	20	10	81	No change
6	TEMPO	1	10	81	No change
7	TEMPO	5	10	81	No change
8	TEMPO	10	10	81	No change
9	TEMPO	20	10	81	No change

**Fig. 6:**
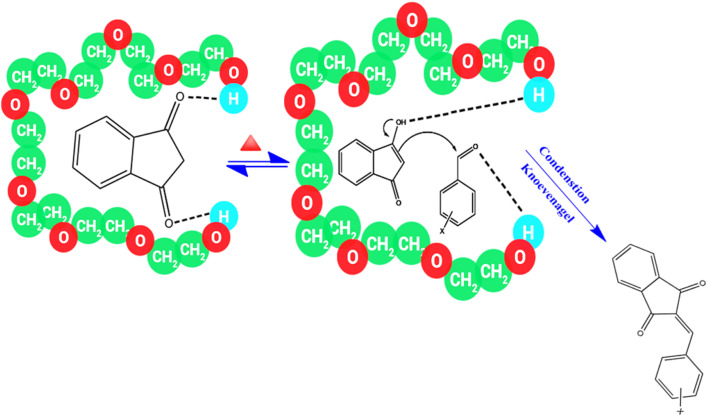
Proposed mechanism for the synthesis of 2-benzylidene-indan-1,3-dione derivatives.

The proposed role of PEG in the synthesis of BZI derivatives can be rationalized based on its unique structural and physicochemical properties. The flexible helical conformation of PEG generates a pseudo-micellar microenvironment that effectively concentrates the reactants, thereby enhancing their mutual proximity and facilitating productive molecular interactions. Activation of the substrates occurs through hydrogen-bonding interactions between the ether oxygen atoms and terminal hydroxyl groups of PEG and the reactive centers of the aldehyde and 1*H*-indene-1,3(2H)-dione. Consequently, PEG functions not only as an environmentally benign solvent but also as a pseudo-catalyst, promoting the Knoevenagel condensation reaction. The reaction proceeds *via* a classical mechanism involving nucleophilic attack of the activated 1*H*-indene-1,3(2H)-dione on the aldehyde carbonyl, followed by dehydration to form the conjugated C=C bond and afford the desired BZI product. The proposed reaction mechanism is illustrated in Fig. 6.

### 3.5 Green chemistry metrics

**Fig. 7:**
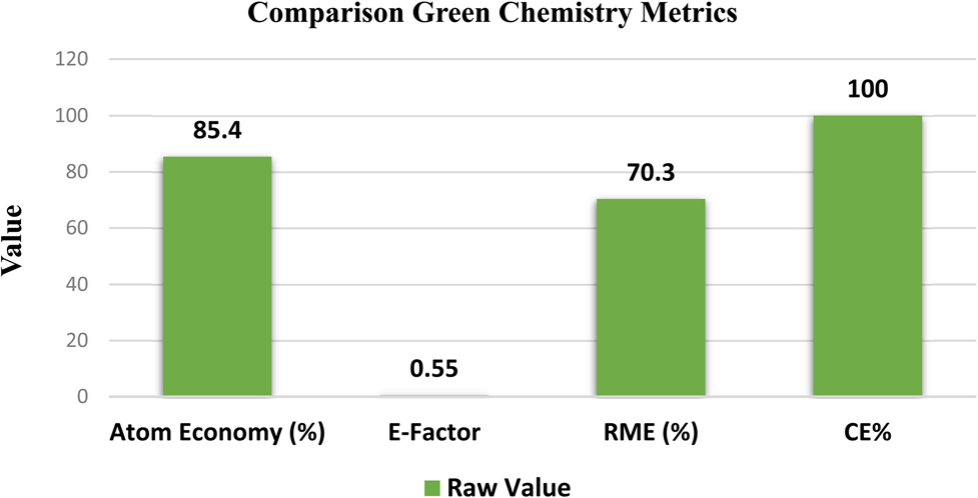
Green chemistry metrics for the synthesis of 2-(4-chlorobenzylidene)-1*H*-indene-1,3(2H)-dione under CSR.

The green chemistry metrics calculated for the CSR-assisted synthesis of 2-(4-chlorobenzylidene)-1*H*-indene-1,3(2H)-dione underscore the environmental sustainability and operational efficiency of the developed protocol. An Atom Economy of 85.4% and a Carbon Efficiency of 100% indicate efficient utilization of reactant atoms in product formation. The low E-factor of 0.55 reflects minimal waste generation during the process. Additionally, the RME of 70.3% demonstrates substantial conversion of starting materials into the desired product. Collectively, these metrics, summarized in Fig. 7, confirm the protocol's strong alignment with green chemistry principles, particularly in waste minimization, resource efficiency, and solvent sustainability.

## 4. Conclusion

In this study, an efficient and sustainable protocol was developed for the synthesis of BZI derivatives by utilizing CSR as a clean, renewable, and abundant thermal energy source. This approach effectively eliminates the need for conventional electrical heating, thereby significantly reducing energy consumption and minimizing environmental impact in accordance with green chemistry principles. The reactions were performed under catalyst-free conditions using PEG-400 as a green solvent, which offers biodegradability, low volatility, and high thermal stability, promoting molecular interactions that facilitate the reaction. The developed methodology demonstrated several advantages, including high product yields within significantly shorter reaction times, mild and straightforward operational conditions, and avoidance of hazardous or volatile reagents. Furthermore, seasonal studies confirmed the practical applicability of CSR under natural sunlight, highlighting its potential as a scalable and sustainable alternative for solar-assisted organic synthesis. Overall, this work contributes to the advancement of green chemistry by integrating solar energy utilization with environmentally benign solvents and catalyst-free protocols, providing a promising route towards more sustainable, energy-efficient, and cost-effective organic transformations aligned with the twelve principles of green chemistry.

## Conflicts of interest

The authors declare no conflict of interest, financial or otherwise.

## Abbreviations

MW: Microwave irradiation
BZI: 2-Benzylidene-indan-1,3-dione
PEG: Polyethylene glycol
PEG-400: Polyethylene glycol 400
IR: Infrared
CSR: Concentrated solar radiation
TLC: Thin-layer chromatography
PDR: Parabolic dish reflector
AE: Atom economy
CE: Carbon efficiency
RME: Reaction mass efficiency

## Supplementary Material

RA-016-D5RA08259E-s001

## Data Availability

The data that support the findings of this study are available from the corresponding author upon reasonable request. Supplementary information (SI) is available. See DOI: https://doi.org/10.1039/d5ra08259e.
