# Supplementary material for: Green and sustainable catalyst-free synthesis of 2-benzylidene-indan-1,3-dione derivatives using concentrated solar radiation in polyethylene glycol
Source: RSC Adv. 2026 Feb 5;16(9):7659–72. doi: 10.1039/d5ra08259e (PMC12875376; doi:10.1039/d5ra08259e)
Supplement: RA-016-D5RA08259E-s001 [file RA-016-D5RA08259E-s001.pdf]

Supplementary Information (SI) for RSC Advances.
This journal is © The Royal Society of Chemistry 2026

Table S1. Recorded weights of starting materials, isolated products, and corresponding yields for all synthesized compounds.

Entry	Product	MW (g/mol)	Weight of Aldehyde (mg)	Weight of 1H-indene-1,3(2H)-dione (mg)	Weight of Product (mg)	Yield (%)
1	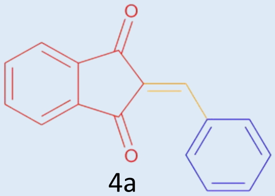 4a	**234**	**127.4**	**146**	**182**	**77**
2	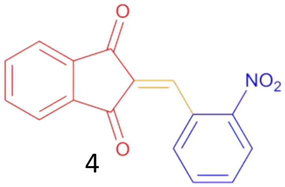 4	**279**	**181.2**	**146**	**255**	**91**
3	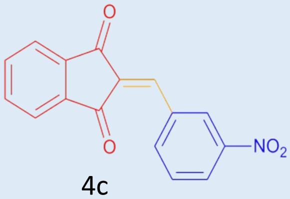 4c	**279**	**181.2**	**146**	**258**	**93**
4	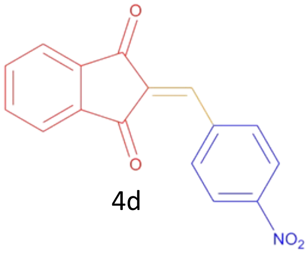 4d	**279**	**181.2**	**146**	**262**	**94**
5	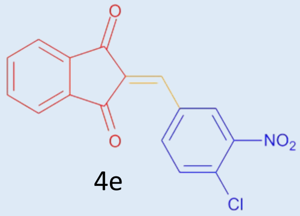 4e	**313**	**222.6**	**146**	**304**	**97**

6	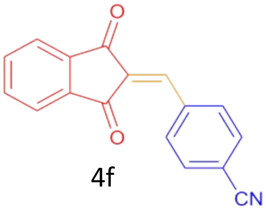 4f	**259**	**157.2**	**146**	**239**	**92**
7	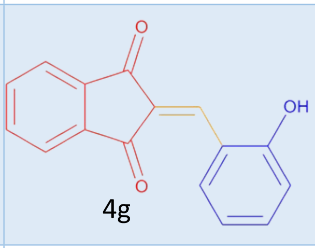 4g	**250**	**146.4**	**146**	**230**	**92**
8	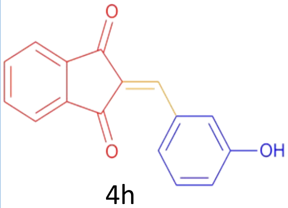 4h	**250**	**146.4**	**146**	**201**	**80**
9	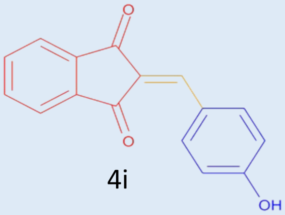 4i	**250**	**146.4**	**146**	**205**	**82**
10	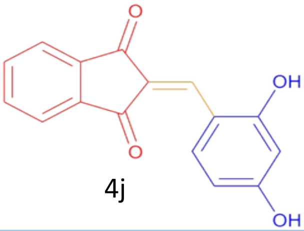 4j	**266**	**165.6**	**146**	**235**	**88**
11	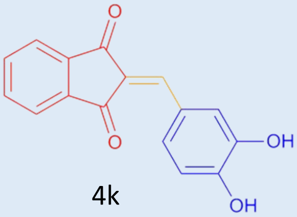 4k	**266**	**165.6**	**146**	**229**	**86**

12	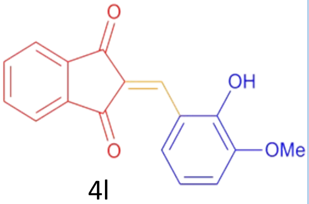 4l	**280**	**182.4**	**146**	**247**	**88**
13	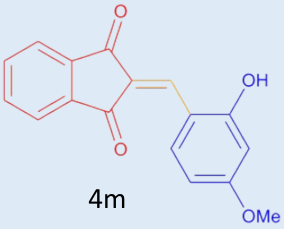 4m	**280**	**182.4**	**146**	**239**	**85**
14	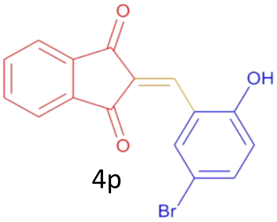 4p	**329**	**241.2**	**146**	**322**	**98**
15	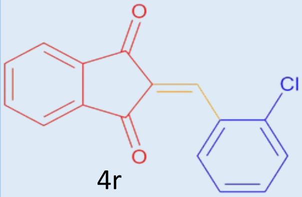 4r	**268**	**168**	**146**	**212**	**79**
16	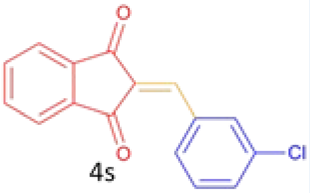 4s	**268**	**168**	**146**	**218**	**81**
17	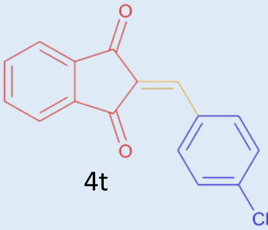 4t	**268**	**168**	**146**	**217**	**81**
18	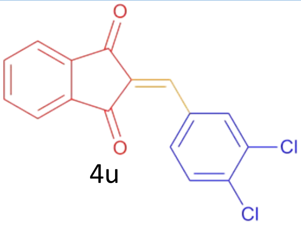 4u	**303**	**210**	**146**	**234**	**77**

19	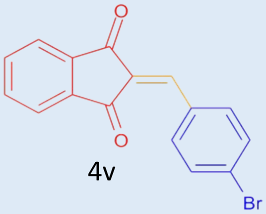 4v	**313**	**222**	**146**	**251**	**80**
20	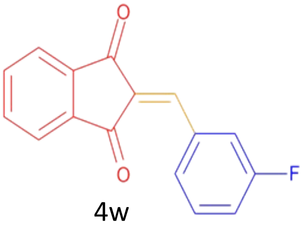 4w	**252**	**148.8**	**146**	**195**	**77**
21	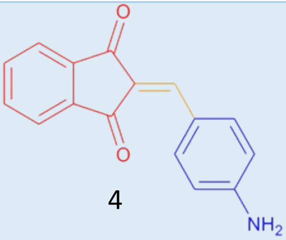 4	**249**	**145.2**	**146**	**242**	**97**
22	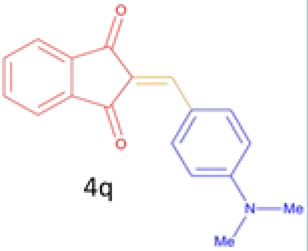 4q	**277**	**180**	**146**	**222**	**80**
23	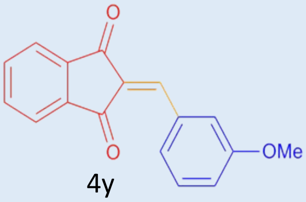 4y	**264**	**163.2**	**146**	**206**	**78**
24	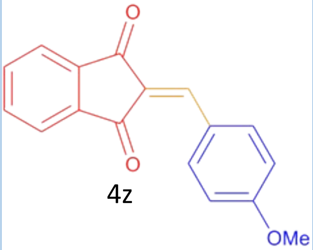 4z	**264**	**163.2**	**146**	**212**	**80**
25	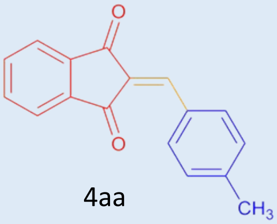 4aa	**248**	**144**	**146**	**191**	**77**

26	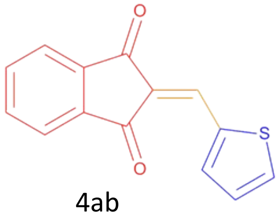 4ab	**240**	**134.4**	**146**	**182**	**76**
27	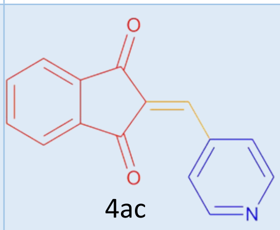 4ac	**235**	**128.4**	**146**	**174**	**74**
28	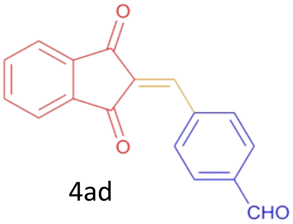 4ad	**262**	**160.1**	**146**	**244**	**93**
29	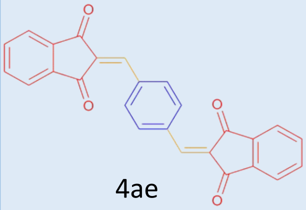 4ae	**390**	**160.1**	**292**	**351**	**91**

Table 2. Recorded solar irradiance, reaction mixture temperatures, and ambient temperatures during all CSR-assisted optimization and substrate-scope reactions.

Entry	Product	irradiance (W/m²)	Temperature of the reaction mixture
1	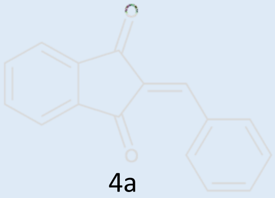 4a	**924**	**128**
2	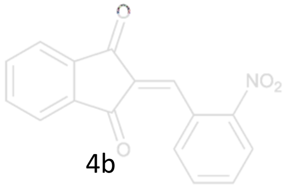 4b	**895**	**127**
3	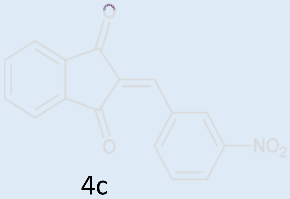 4c	**890**	**125**
4	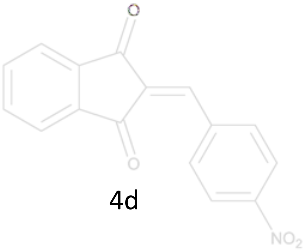 4d	**857**	**134**
5	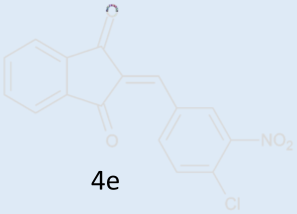 4e	**883**	**131**

6	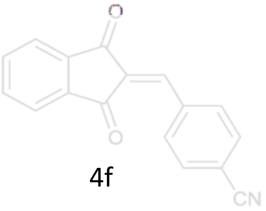 4f	**926**	**128**
7	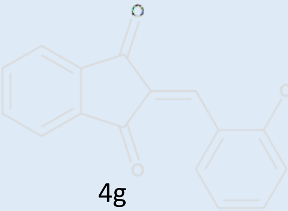 4g	**902**	**139**
8	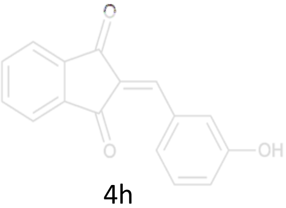 4h	**964**	**131**
9	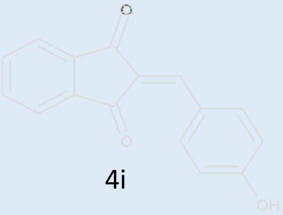 4i	**889**	**141**
10	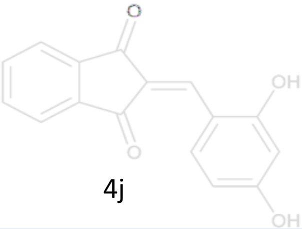 4j	**873**	**134**
11	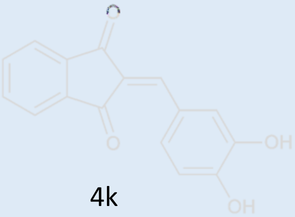 4k	**855**	**128**

12	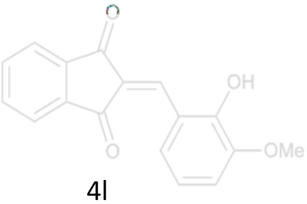 4l	**985**	**95**
13	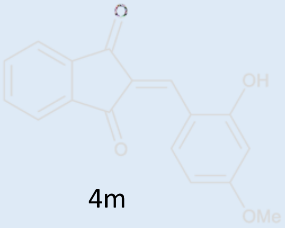 4m	**892**	**122**
14	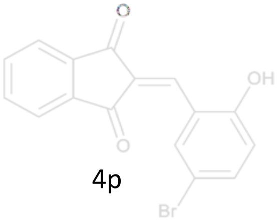 4p	**906**	**126**
15	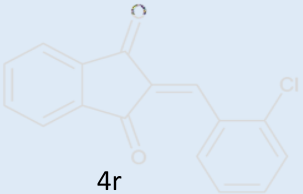 4r	**1000**	**131**
16	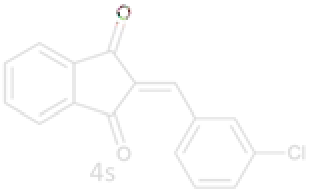 4s	**864**	**138**
17	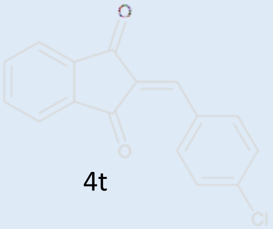 4t	**880**	**136**
18	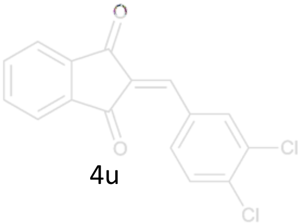 4u	**926**	**134**

19	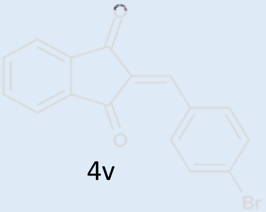 4v	**989**	**134**
20	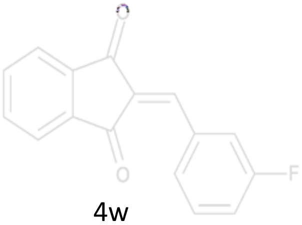 4w	**861**	**126**
21	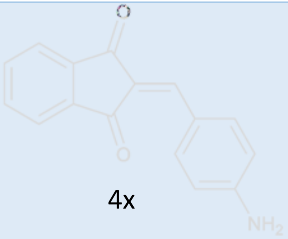 4x	**872**	**145**
22	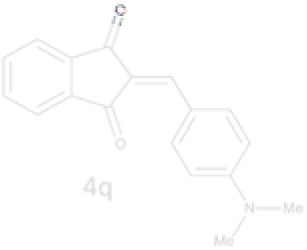 4q	**863**	**160**
23	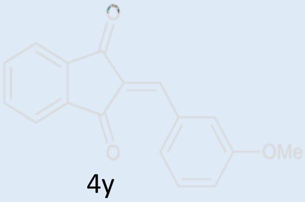 4y	**872**	**127**
24	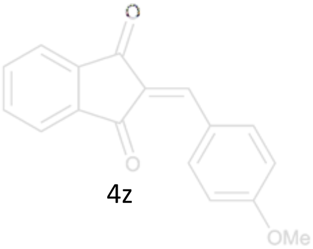 4z	**908**	**150**
25	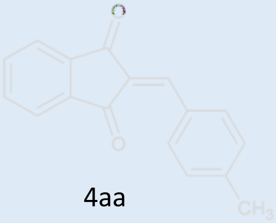 4aa	**938**	**153**

26	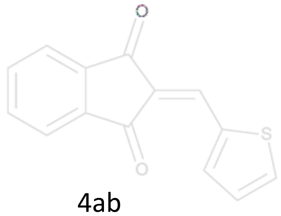 4ab	**911**	**136**
27	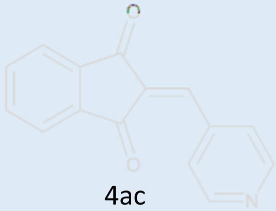 4ac	**871**	**124**
28	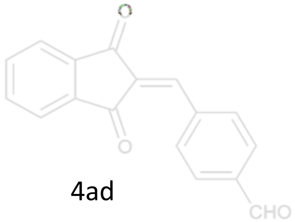 4ad	**925**	**115**
29	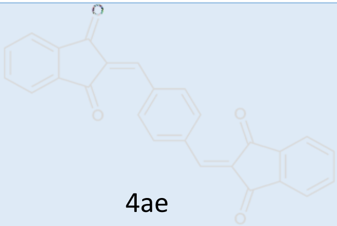 4ae	**923**	**132**

Spectra:

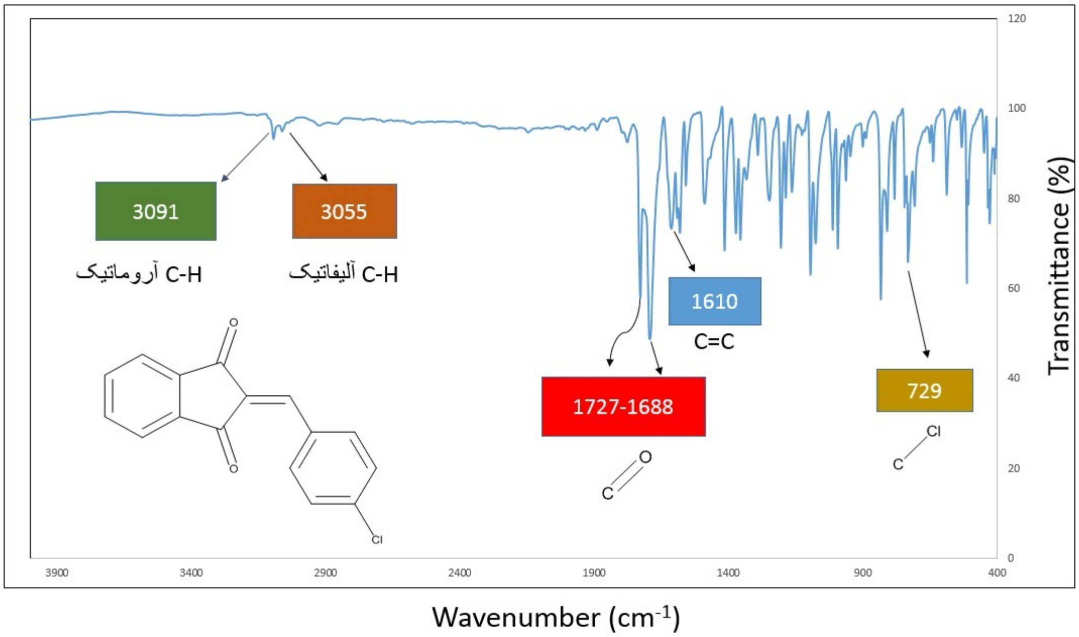

Spectrum 1: FT-IR spectrum of 2-(4-chlorobenzylidene)-2H-indene-1, 3-dione.

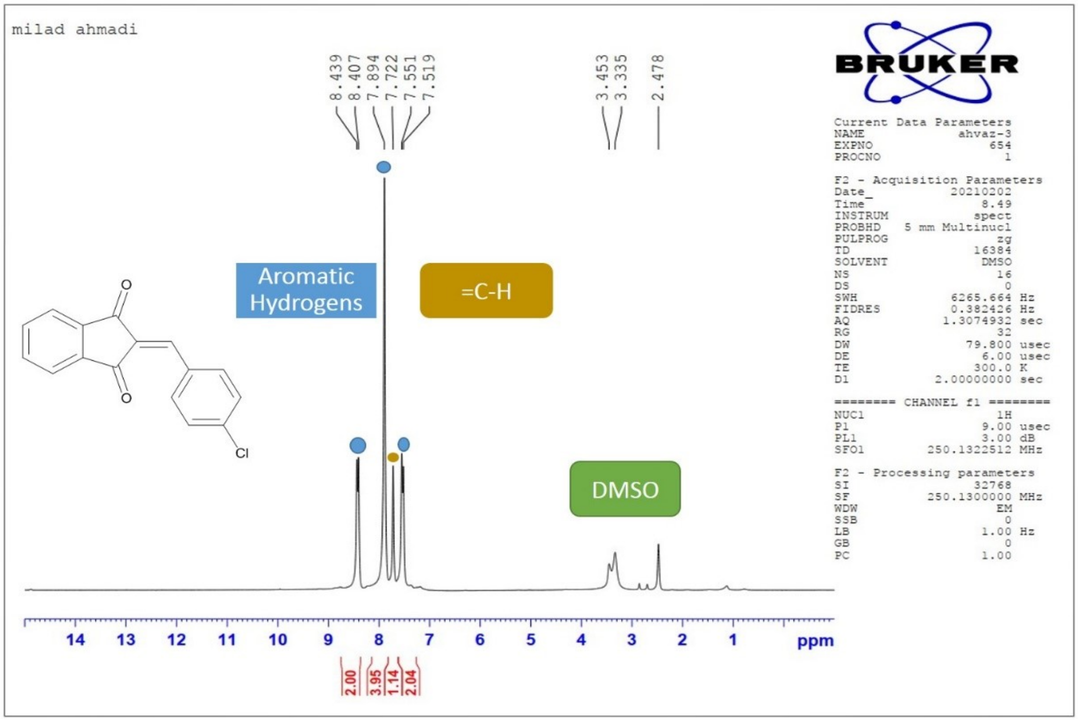

Spectrum 2: ^{1}H NMR spectrum of 2-(4-chlorobenzylidene)-2H-indene-1, 3-dione.

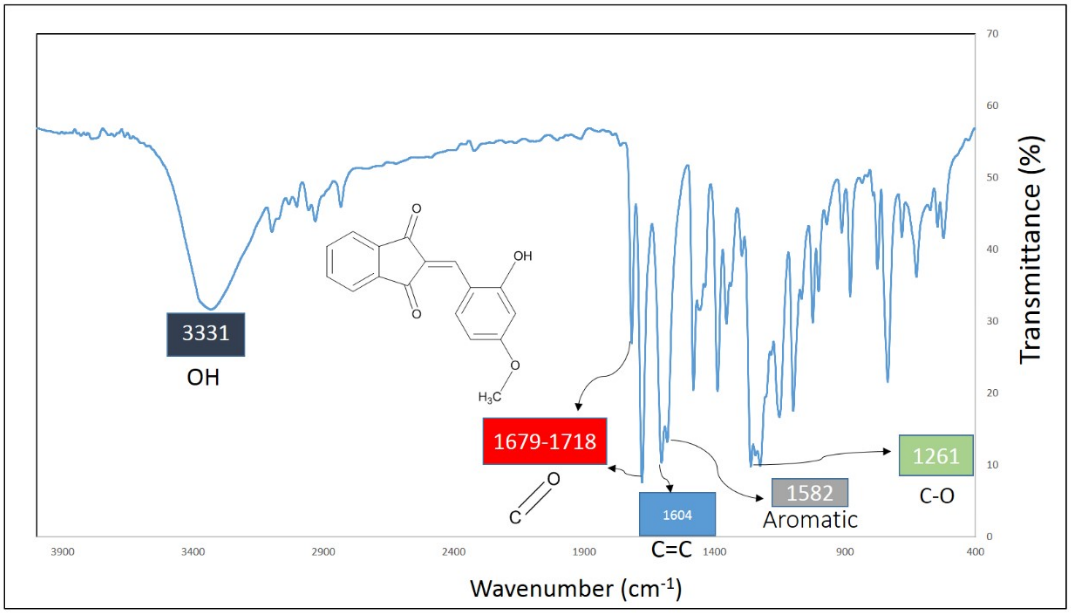

Spectrum 3: FT-IR spectrum of 2-(2-hydroxy- 4-methoxybenzylidene)-2H-indene-1, 3-dione.

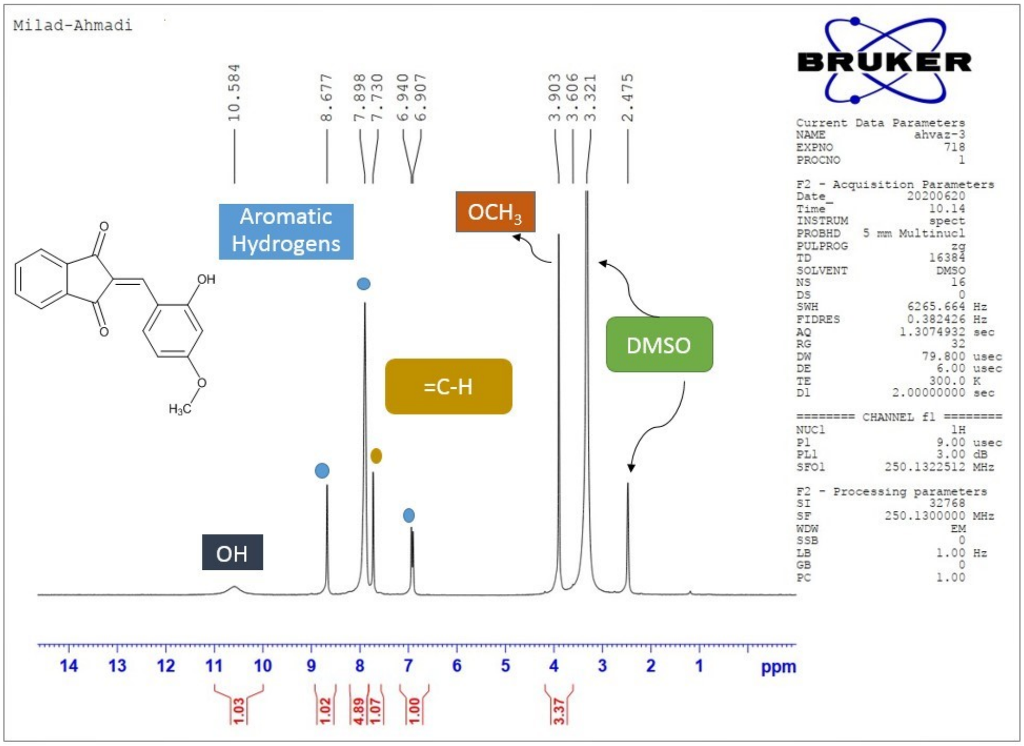

Spectrum 4: The ^{1}H NMR spectrum of 2-(2-hydroxy- 4-methoxybenzylidene)-2H-indene-1, 3-dione.

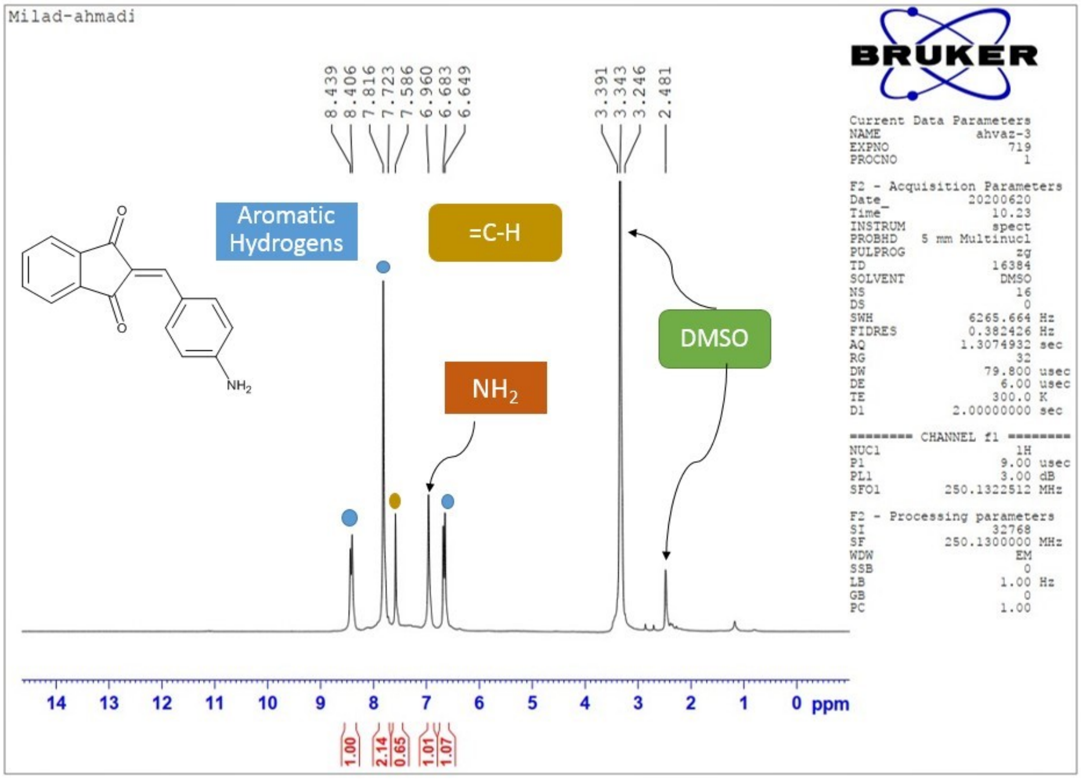

Spectrum 5: The ^{1}H NMR spectrum of 2-(4-aminobenzylidene)-2H-indene-1,3-dione.
